# Supplementary material for: Seroprevalence of brucellosis in small ruminants and related risk behaviours among humans in different husbandry systems in Mali
Source: PLoS One. 2021 Jan 22;16(1):e0245283. doi: 10.1371/journal.pone.0245283 (PMC7822284; doi:10.1371/journal.pone.0245283)
Supplement: S1 File — (PDF) [file pone.0245283.s001.pdf]

FREE AND INFORMED ASSENT FORM

Project title : Role of small ruminant husbandry systems in the transmission of brucellosis to animals and humans in Mali.

Declaration of the minor's tutor:
In my capacity as legal representative, I have read and understood the contents of this form. I certify that it was explained to me verbally. I had the opportunity to ask all my questions and they were answered to my satisfaction. I know that I am free to accept that my child (or the person I represent) participate in the project just as I am free to withdraw him (her) from the project at any time, by verbal notice, without prejudice. I certify that we have been given sufficient time to make our decision. I also certify that my child (or the person I represent) does not object. I will receive a signed and dated copy of this form. I, the undersigned, consent to my child (or the person I represent) participating in the project.

Name of parent or legal tutor : ____
Place : _____________ Date : _____________ Signature : _______________________
Name of minor : ____
Place : _____________ Date : _____________ Signature : _______________________
Name of witness if applicable : ____
Place : _____________ Date : _____________ Signature : _______________________

--- Thank you for your participation ! -------------------------------------

---

FREE AND INFORMED ASSENT FORM

Project title : Role of small ruminant husbandry systems in the transmission of brucellosis to animals and humans in Mali.

Declaration of the minor's tutor:
In my capacity as legal representative, I have read and understood the contents of this form. I certify that it was explained to me verbally. I had the opportunity to ask all my questions and they were answered to my satisfaction. I know that I am free to accept that my child (or the person I represent) participate in the project just as I am free to withdraw him (her) from the project at any time, by verbal notice, without prejudice. I certify that we have been given sufficient time to make our decision. I also certify that my child (or the person I represent) does not object. I will receive a signed and dated copy of this form. I, the undersigned, consent to my child (or the person I represent) participating in the project.

Name of parent or legal tutor : ____
Place : _____________ Date : _____________ Signature : _______________________
Name of minor : ____
Place : _____________ Date : _____________ Signature : _______________________
Name of witness if applicable : ____
Place : _____________ Date : _____________ Signature : _______________________

--- Thank you for your participation ! -------------------------------------
