# Supplementary material for: Seroprevalence of brucellosis in small ruminants and related risk behaviours among humans in different husbandry systems in Mali
Source: PLoS One. 2021 Jan 22;16(1):e0245283. doi: 10.1371/journal.pone.0245283 (PMC7822284; doi:10.1371/journal.pone.0245283)
Supplement: S2 File — (PDF) [file pone.0245283.s002.pdf]

INFORMATION

Project title: Role of small ruminant husbandry systems in the transmission of brucellosis to animals and humans in the regions of Sikasso, Ségou and the district of Bamako in Mali.

Identity of researchers and partner institutions: Ecole Inter-Etats des Sciences et Médecine Vétérinaire de Dakar : Dr. S. TRAORE. Prof. R. BADA ALAMBEDJI, Laboratoire Centrale Vétérinaire de Bamako : Dr. K. COUIBALY. Afrique One ASPIRE : Prof. B. Bonfoh, Dr. G. FOKOU. SUA-Tanzanie : R. KAZWALA

Objectifs de l'étude : We would like to include your herd in our study which aims to determine the role of husbandry systems in the transmission of brucellosis to animals and humans in Mali. It will take place in several villages in your area. This infection can cause serious health problems for you and your animals, such as recurrent fevers, reduced milk and meat production. Herd abortions and breeding failures. The information gathered will help in the planning and implementation of future interventions to overcome this disease in the region and in your village and if possible will help the entire population of Mali.

Procédures de recherche:

Serologicals tests: In our study, we would like to know the number of cases of infections due to the bacteria responsible for brucellosis in small ruminants in your herd. For this reason, we will need blood samples (5ml) from some of your animals at least 6 months of age. The blood samples will be sent to the LCV Medical Bacteriology laboratory before being transported to the EISMV Microbiology laboratory in Dakar. They will be examined for the presence of bacteria responsible for brucellosis. We look forward to your participation and / or anyone looking after the herd.

Questionnaire survey: The objective of this part of the study is to identify the attitudes and practices of the communities in relation to the health situation. We have designed a questionnaire for this purpose and we would like to invite you to participate. The questionnaire could take between 15-20 minutes to answer our questions.

Confidentiality: All information collected in this study will be encoded and stored in a secure location. Blood samples will be labeled with these codes and stored in a secure location for testing. Only study members will have access to the data. Members of the INRSP - BAMAKO ethics committee will have access to the information collected to monitor good clinical practice. Study participants will also have access to the information collected. We will publish the salient results of this study but your name and identity will not be revealed.

Consent: Your participation or that of your employee in this study is entirely voluntary even after having signed the consent form. There is no obligation to participate in this study.

Alternative participation: If you decide not to participate, it will not affect relationships with team members in any way.

Consequences if you decide to withdraw from the study and the methodological procedure before the end of your participation: You can decide to withdraw from the study at any time and without prejudice. However, we would like to inform you that the data collected prior to your withdrawal may be used for reports and publications.

Any other concerns?

Please do not hesitate to contact us if you have any further concerns at the following address:

Dr. Souleymane TRAORE (Chercheur principal), EISMV-DAKAR BP 5077 – Dakar Fann - Sénégal
Tél. : +223 78 84 35 70 / +221 77 353 37 05, souleymanot@yahoo.fr
Madame Sidibé Diaba Camara (Vice-présidente, Comité éthique INRSP, Tel : 66 76 63 37
Téléphone secrétariat de l'INRSP : +223 20 21 43 20

FREE AND INFORMED CONSENT FORM

Project title : Role of small ruminant husbandry systems in the transmission of brucellosis to animals and humans in the regions of Sikasso, Ségou and the district of Bamako in Mali.

Participant's declaration:

☐ I have read the study information sheet
☐ the study information sheet was read to me
☐ the study information sheet has been translated into a language I understand.

I had the opportunity to ask questions and all my concerns were addressed. I know the purpose, objectives and procedures. I understand that I can withdraw from the study at any time without any consequences. I have received a copy of this informed consent form and an additional information sheet that I am maintaining.

Participant's name: ____

Place : ____________ Date : ____________ Signature : ______________________

If the participant cannot read

I witnessed the precise reading of the consent form to the potential participant and the individual had the opportunity to ask questions. I confirm that the person has given their consent freely.

Name of witness if applicable: ____

Place : ____________ Date : ____________ Signature : ______________________

-- Thank you for your participation! --------------------------------------

---

FREE AND INFORMED CONSENT FORM

Project title : Role of small ruminant husbandry systems in the transmission of brucellosis to animals and humans in the regions of Sikasso, Ségou and the district of Bamako in Mali.

Participant's declaration:

☐ I have read the study information sheet
☐ the study information sheet was read to me
☐ the study information sheet has been translated into a language I understand.

I had the opportunity to ask questions and all my concerns were addressed. I know the purpose, objectives and procedures. I understand that I can withdraw from the study at any time without any consequences. I have received a copy of this informed consent form and an additional information sheet that I am maintaining.

Participant's name: ____

Place : ____________ Date : ____________ Signature : ______________________

If the participant cannot read

I witnessed the precise reading of the consent form to the potential participant and the individual had the opportunity to ask questions. I confirm that the person has given their consent freely.

Name of witness if applicable: ____

Place : ____________ Date : ____________ Signature : ______________________

-- Thank you for your participation! --------------------------------------
