# Supplementary material for: Seroprevalence of brucellosis in small ruminants and related risk behaviours among humans in different husbandry systems in Mali
Source: PLoS One. 2021 Jan 22;16(1):e0245283. doi: 10.1371/journal.pone.0245283 (PMC7822284; doi:10.1371/journal.pone.0245283)
Supplement: S3 File — (PDF) [file pone.0245283.s003.pdf]

Questionnaire sur le cheptel et les comportements à risque

Merci de nous accorder un peu de votre temps afin de repondre à nos questions

1. Number

2. Date

3. Region
○ 1. Bamako ○ 2. Sikasso ○ 3. Segou

4. Locality

5. GPS coordinates

Identification of the respondent

6. What is your name please ?

7. What age group are you in?
○ 1. [0 18] ○ 2. [18 25] ○ 3. [25 45] ○ 4. [45 ..[

8. Gender
○ 1. Male ○ 2. female

9. What is your marital status?
○ 1. Single ○ 2. Maried ○ 3. Widower ○ 4. Divorced

10. Are you educated
○ 1. yes ○ 2. no

11. If yes, what type of school did you attend ?
○ 1. Koranic ○ 2. French ○ 3. Local
La question n'est pertinente que si Alphabetisation = "yes"

12. What is your education level ?
○ 1. Basic ○ 2. Secondary ○ 3. High
La question n'est pertinente que si Alphabetisation = "yes"

13. What is your main professional activity?
○ 1. Breeding ○ 2. Agriculture ○ 3. Other

14. If other activity specify ?

La question n'est pertinente que si Activite = "Other"

Characterization of the farm

15. How far is the farm from the main urban center?
○ 1. [0 24] ○ 2. [25 50] ○ 3. [50 ..[

16. Do you own a herd of?
○ 1. ovine ○ 2. Caprine ○ 3. Ovine+caprine

17. Are there other species besides small ruminants?
○ 1. yes ○ 2. no

18. If yes which?
☐ 1. ovine ☐ 2. Poultry ☐ 3. Equidae
☐ 4. pigs ☐ 5. Others
Vous pouvez cocher plusieurs cases (4 au maximum).
La question n'est pertinente que si Autre_espèces = "yes"

19. Do the animals live in an enclosure?
○ 1. yes ○ 2. no

20. If yes what kind of material is the enclosure made of?
○ 1. cement ○ 2. Banco ○ 3. wood ○ 4. fences
○ 5. Iron bar ○ 6. Stake
La question n'est pertinente que si Enclos = "yes"

21. How did you acquire your herd ?
○ 1. Purchase ○ 2. Don ○ 3. Heritage

22. Is the herd in?
○ 1. Unique ○ 2. Mixed

23. What is the goal of your breeding?
○ 1. Milk ○ 2. Fattening ○ 3. Prestige ○ 4. Income

24. How many heads do you think you have?

25. Alimentation mode ?
○ 1. Give all ○ 2. Just_supplement ○ 3. Only grazing

26. Medical care for animals ?
○ 1. Nothing ○ 2. Maladie ○ 3. regularly

27. Do your animals movements
○ 1. yes ○ 2. no

28. If yes, how would you describe the movements of your herd?
○ 1. Daily ○ 2. Seasonal ○ 3. annual
La question n'est pertinente que si Deplacement = "yes"

29. What are your reasons for moving the herd?
○ 1. Pature ○ 2. vaccination ○ 3. other
La question n'est pertinente que si Deplacement = "yes"

30. If other, specify ?

La question n'est pertinente que si Raison_deplacement = "other"

31. Does your herd come into contact with other herds?

○ 1. yes ○ 2. no

Risk behaviors

32. Are you in constant contact with your animals?

○ 1. yes ○ 2. no

33. If so, what is the level of proximity to animals?

☐ 1. Sleep with animal ☐ 2. Enclosure_in_concession
☐ 3. Animal_aroud_house ☐ 4. Milked
☐ 5. Treatments ☐ 6. Grazing

Vous pouvez cocher plusieurs cases (3 au maximum).
La question n'est pertinente que si Contact_permanent = "yes"

34. What breeding method (s) do you use?

○ 1. SN ○ 2. IA ○ 3. SN+IA

35. If natural rearing, do you trade breeding males with other farms?

○ 1. yes ○ 2. no

La question n'est pertinente que si Reproduction = "SN"

36. Are your animals milked?

○ 1. yes ○ 2. no

37. If so, what method of milking do you use?

○ 1. Manual ○ 2. Mechanical

La question n'est pertinente que si Traite = "yes"

38. If manual, how are the hands of the person milking the animals?

○ 1. With gloves ○ 2. Without gloves

La question n'est pertinente que si Methode_Traite = "Manual"

39. What do you do with the collected milk?

☐ 1. Consumes ☐ 2. Sold ☐ 3. Share

Vous pouvez cocher plusieurs cases.
La question n'est pertinente que si Traite = "yes"

40. If consumed, in what state do you consume this milk?

○ 1. Raw ○ 2. Boiled ○ 3. Fermented

La question n'est pertinente que si Lait_recueilli = "Consumes"

41. Do you assist the females during calving?

○ 1. Yes ○ 2. no

42. If yes, How are the hands of the person helping the animals to give birth?

○ 1. With gloves ○ 2. Without gloves

La question n'est pertinente que si Aide_mise_bas = "Yes"

43. What do you do with the placentas?

○ 1. Away ○ 2. Bury ○ 3. Burn ○ 4. Others

44. If other, specify?

La question n'est pertinente que si Gestion_Placentas = "Others"

45. Do you have any cases of hygromas in your herd?

○ 1. yes ○ 2. no

46. If yes, what do you do with these animals

○ 1. Solde ○ 2. Keep ○ 3. slaughter

La question n'est pertinente que si Hygrogroma = "yes"

47. Do you have any cases of abortions in your herd?

○ 1. yes ○ 2. no

48. If so, what do you do with these animals?

○ 1. Solde ○ 2. Keep ○ 3. slaughter

La question n'est pertinente que si Avortements = "yes"

49. If yes, how do you deal with abortions?

○ 1. Away ○ 2. Bury ○ 3. To dogs ○ 4. Others

La question n'est pertinente que si Avortements = "yes"
