# Supplementary material for: Seroprevalence of brucellosis in small ruminants and related risk behaviours among humans in different husbandry systems in Mali
Source: PLoS One. 2021 Jan 22;16(1):e0245283. doi: 10.1371/journal.pone.0245283 (PMC7822284; doi:10.1371/journal.pone.0245283)
Supplement: S4 File — (PDF) [file pone.0245283.s004.pdf]

Questionnaire sur le cheptel et les comportements à risque

Merci de nous accorder un peu de votre temps afin de repondre à nos questions

1. Numero de fiche

2. Date

3. Région
- ○ 1. Bamako ○ 2. Sikasso ○ 3. Segou

4. Localité

5. Coordonnées Geographique

Identification de l'enqêté

6. Quel est votre nom ?

7. Dans quelle tranche d'âge vous situez vous ?
- ○ 1. Moins de 18 ans ○ 2. De 18 à 25 ans
- ○ 3. De 25 à 45 ans ○ 4. Plus de 45 ans

8. Genre
- ○ 1. Masculin ○ 2. Féminin

9. Quelle est votre situation matrimoniale ?
- ○ 1. Célibataire ○ 2. Marié(e) ○ 3. Veuf(ve)
- ○ 4. Divorcé(e)

10. Etes vous instruit(e)
- ○ 1. oui ○ 2. non

11. Si oui, Quelle type d'école avez vous frequeté
- ○ 1. Ecole coranique ○ 2. Ecole française
- ○ 3. Langue locale

La question n'est pertinente que si Alphabetisation = "oui"

12. Quel est votre niveau d'étude
- ○ 1. Basique ○ 2. Moyen ○ 3. Elevé

La question n'est pertinente que si Alphabetisation = "oui"

13. Quelle est votre activité professionnelle principale ?
- ○ 1. Elevage ○ 2. Agriculture ○ 3. Autre

14. Si autre activité precisez

Caracterisation de l'élevage

15. L'élevage est à quelle distance du principal centre urbain
- ○ 1. Entre 0 - 24 Km ○ 2. Entre 25 - 50 Km
- ○ 3. Au-delà de 50 Km

16. Vous possedez un troupeau de
- ○ 1. Ovins ○ 2. Caprins ○ 3. Ovins+Caprins

17. Il y a d'autres èspèces en plus des petits ruminants ?
- ○ 1. oui ○ 2. non

18. Si oui lesquelles?
- ☐ 1. Bovins ☐ 2. volaille ☐ 3. Equidés
- ☐ 4. porcins ☐ 5. autres

Vous pouvez cocher plusieurs cases (4 au maximum).

19. Comment avez vous acquis votre troupeau
- ○ 1. Achat ○ 2. Don ○ 3. Héritage

20. Le troupeau est il en ?
- ○ 1. Propriété unique ○ 2. Propriété mixte

21. Quelle est le but de votre élevage
- ☐ 1. Production laitière ☐ 2. Embouche
- ☐ 3. Elevage de prestige ☐ 4. Activité culturelle

Vous pouvez cocher plusieurs cases (3 au maximum).

22. Combien de têtes estimez-vous posséder ?

23. Mode d'alimentation
- ○ 1. Donne tout le nécessaire aux animaux sur place
- ○ 2. Donne juste des suppléments aux animaux
- ○ 3. Animaux amenés sur le pâturage

24. Soins médicaux apportés aux animaux
- ☐ 1. N'apporte pas de soins
- ☐ 2. Seulement en cas de maladie
- ☐ 3. Suivi régulier par un professionnel de la santé animale

Vous pouvez cocher plusieurs cases.

25. Est ce que vos animaux effectuent des deplacements
- ○ 1. Oui ○ 2. Non

26. Si oui, Comment décririez-vous les mouvements de votre troupeau ?
- ○ 1. Aller-retour dans la même journée
- ○ 2. Longue distance parcourue au gré des saisons
- ○ 3. Mouvement tout au long de l'année

La question n'est pertinente que si Mouvement aniamux = "Oui"

27. Pour quelles raisons deplacez vous le troupeau ?

- ○ 1. Recherche de pâturage et d'eau
- ○ 2. Campagne de vaccination
- ○ 3. Rencontre annuelles
- ○ 4. Autre

28. Si autre précisez

29. Ces déplacements ont lieu à quelle periode de l'année ?

- ○ 1. Saison sèche froide ○ 2. Saison sèche chaude
- ○ 3. Saison pluvieuse

30. Votre troupeau rentre t'il en contact avec d'autres troupeau ?

○ 1. oui ○ 2. non

31. Si oui, quel type de contact ?

- ☐ 1. Sur pâturage
- ☐ 2. Sur les points d'abreuvement
- ☐ 3. Dans les parcs de vaccination
- ☐ 4. En confiant la garde du troupeau
- ☐ 5. Pendant les prêts d'animaux

Vous pouvez cocher plusieurs cases (3 au maximum).

Comportements à risque

32. Etes-vous en contact permanent avec vos animaux ?

○ 1. oui ○ 2. non

33. Si oui; quelle est le niveau de proximité avec les animaux

- ☐ 1. Dort avec les animaux
- ☐ 2. Enclos dans la concession
- ☐ 3. Animaux dispersés autour de la maison
- ☐ 4. lors de la traite
- ☐ 5. pendant les soins
- ☐ 6. lors de la conduite de animaux

Vous pouvez cocher plusieurs cases (3 au maximum).

34. Quelle(s) méthode(s) de reproduction utilisez-vous

- ○ 1. saillie naturelle
- ○ 2. Insémination Artificielle
- ○ 3. saillie naturelle + Insémination artificielle

35. Si monte naturelle, est-ce que vous faites des échanges de males reproducteurs avec d'autres fermes ?

○ 1. oui ○ 2. non

36. Est ce que vos animaux sont traits

○ 1. Oui ○ 2. non

37. Si oui, quelle methode de traite utilisez vous ?

○ 1. manuelle ○ 2. mecanique

La question n'est pertinente que si traite = "Oui"

38. Si manuelle, comment sont les mains de la personne qui trait les animaux ?

○ 1. Avec gants ○ 2. sans gants

39. Que faites vous du lait recueilli

○ 1. Consomme ○ 2. vend ○ 3. partage

40. Si consommé, dans quel état consommez vous ce lait ?

- ○ 1. consommé cru ○ 2. bouilli avant
- ○ 3. fermenté avant

41. Assistez-vous les femelles pendant les mises bas ?

○ 1. oui ○ 2. non

42. Comment sont les mains de la personne qui aide les animaux à mettre bas?

○ 1. Gantée ○ 2. Non Gantée

43. Que faites-vous du placentas ?

- ☐ 1. Jetter hors ferme ☐ 2. Enterrer ☐ 3. Bruler
- ☐ 4. Autre

Vous pouvez cocher plusieurs cases (3 au maximum).

44. Si autres, précisez

45. Avez-vous des cas d'hygromas dans votre troupeau ?

○ 1. Oui ○ 2. Non

46. Si oui que faites- vous de ces animaux

○ 1. Vendre ○ 2. garder ○ 3. abattre

47. Avez-vous des cas d'avortements dans votre troupeau ?

○ 1. Oui ○ 2. Non

48. Si oui que faites-vous de ces animaux ?

○ 1. Vendre ○ 2. garder ○ 3. Abattre

49. Si oui, comment gérez-vous les avortons ?

- ○ 1. On les jette ○ 2. On les enterre
- ○ 3. On les laisse aux chiens ○ 4. Autre

La question n'est pertinente que si Avortements = "Oui"
