# Supplementary material for: Seroprevalence of brucellosis in small ruminants and related risk behaviours among humans in different husbandry systems in Mali
Source: PLoS One. 2021 Jan 22;16(1):e0245283. doi: 10.1371/journal.pone.0245283 (PMC7822284; doi:10.1371/journal.pone.0245283)
Supplement: Attachment — Submitted filename: Response to Reviewers.docx [file pone.0245283.s008.docx]

Point-by-point response (see the changes in the manuscript file with track changes).

#Editor

- Please address the following: - Please confirm that parental consent was not only requested, but obtained for participants who were minors.

The consent of each minor interviewed was obtained in addition to that of the responsible adult (see consent form attached). The statement has been changed to reflect that the consent for participants who were minors was actually obtained Line [205]

- Please include additional information regarding the survey or questionnaire used in the study and ensure that you have provided sufficient details that others could replicate the analyses. For instance, if you developed a questionnaire as part of this study and it is not under a copyright more restrictive than CC-BY, please include a copy, in both the original language and English, as Supporting Information.

English and French versions of the questionnaire have been attached (see the attached file)

- In your Methods section, please provide additional information about the demographic details of your participants. Please ensure you have provided sufficient details to replicate the analyses such as: a) a description of any inclusion/exclusion criteria that were applied to participant inclusion in the analysis, b) a table of relevant demographic details, c) a statement as to whether your sample can be considered representative of a larger population.

a. Inclusion / exclusion criteria are describe in the methods section line [98 - 101]
b. Demographic details: Demographic details about study participants have been added in the results section. line [204 - 213]
c. Sample representativeness: The sample can be confidently considered as representative of a larger population because we used random sampling method at all levels.

- Please ensure you have thoroughly discussed any potential limitations of this study within the Discussion section.

The limitations of the study are discussed in the discussion section. Line [331 - 339]

- Please state the dates during which data collection took place.

Month and dates of the study are include in the ‘’methods section’’ line [75]

- We note that you have indicated that data from this study are available upon request. PLOS only allows data to be available upon request if there are legal or ethical restrictions on sharing data publicly. For information on unacceptable data access restrictions, please see <http://journals.plos.org/plosone/s/data-availability#loc-unacceptable-data-access-restrictions>.

The data are attached with the files associated with the manuscript

Reviewer #1: Manuscript Number: PONE-D-20-21180 Title: “Seroprevalence of brucellosis in small ruminants and associated risk behaviours for humans in different husbandry systems in Mali” I appreciate the efforts by the authors to address the role of small ruminant husbandry systems in maintaining and transmitting brucellosis in Mali. Brucellosis remain an important endemic zoonoses in many of the developing nations, where such epidemiological studies need to be carried out to reveal the true prevalence and risk factors for the disease. I have major concern with the presentation of the results in terms of possible risk factors without the calculation of the measures of association, which I have highlighted in comment number 4.

Comments:

1. Line 53-55: “Livestock in Mali is facing many health concerns and many diseases hinder the development of the production in the country among which brucellosis [3]”…. The sentence seems to be incomplete, rephrase it

The sentence has been rephrased, line [54 - 55]

2. Line 56: “The species *B. melitensis* which is the most widespread in small ruminants seems to be the most pathogenic in humans”: Provide the reference for it.

The references have been added, line [59]

3. Line 106: “In each locality (village, hamlet), six farms were chosen”: The rationale for choosing six farms need to be described.

The rationale has been described Line [106 - 110]

4. Results: The major issue with results are that there is no calculation for measures of association, the authors depicted the results just in % seroprevalence for different parameters. The univariate and multivariate analysis of the possible risk factors need to be estimated to justify the objectives of the study in terms of risk factors.

We performed logistic regression analysis to determine association between the predetermined risk factors and seropositivity. See in the methodology section Line [178 - 183], In the results section line [267 - 278] and in the discussion section.

5. Line 234: “Risk behaviors of brucellosis transmission identified among farmers”: The authors presented the frequency of the possible risk factors for brucellosis transmission on the basis of the responses to the questionnaire. There is also need to find the association with the farm level prevalence of brucellosis. The title is little misnomer, the authors didn’t study the transmission pathways of brucellosis and there is no supporting data for human brucellosis prevalence

In the present study we focussed on the seroprevalence of brucellosis in animals and risk behaviours that would facilitate transmission of the disease/infection within animals and between animals and humans.

One of the limitations of the study is the fact that we did not perform serological tests in humans. The study was limited to the observation of presence of risky behaviours as identified by several authors. Presence of infected animals and risky behaviours are the critical as will facilitate transmission of the disease. The next stage is now to check on the exposure status on the humans.

6. Line 247: “this is an up-to-date study conducted”: The sentence needs to be revised

[the sentence has been revised line 280 - 282].

7. Line 249: We used two diagnostic “technics”: Spell check it

(the sentence has been deleted

8. Line 252: “Seroprevalence and risk behaviors will be the main points around which the discussion will be structured”: Need to be revised.

This sentence has been deleted

9. Line 265-267: “He also argued that a high animal concentration in an area would favour animal infection. This was somewhat confirmed by our results.”: There is simply the mention of herd size and seroprevalence in Table 1, the association between these need to be estimated before arriving any conclusion

We performed a regression analysis and we found the association between herd size and seropositivity [see table 4].

10. Figure 2 (now Figure 1): In Segou, the bar chart needs to be revised to bring urban bar together with rest of the two

The urban bar has been brought closer to the other two bars See Figure 1.

11. Figure 3: Brucellosis risk behaviors identified according to small ruminant’s husbandry systems: I don’t think that this figure provides any significant finding, it can be supplementary Table.

We have removed figure 3 as most of the information is found in the text.
