# Supplementary material for: Seroprevalence of brucellosis in small ruminants and related risk behaviours among humans in different husbandry systems in Mali
Source: PLoS One. 2021 Jan 22;16(1):e0245283. doi: 10.1371/journal.pone.0245283 (PMC7822284; doi:10.1371/journal.pone.0245283)
Supplement: Attachment — Submitted filename: Response to Reviewers.docx [file pone.0245283.s009.docx]

Responses to reviewers

The whole document has been checked, spelling and typographical errors have been revised.
